# Improbable but true: the invasive inbreeding ambrosia beetle *Xylosandrus morigerus* has generalist genotypes

**DOI:** 10.1002/ece3.58

**Published:** 2012-01

**Authors:** Hanne F Andersen, Bjarte H Jordal, Marius Kambestad, Lawrence R Kirkendall

**Affiliations:** 1 Department of Biology, University of BergenBergen, Norway; 2 The Natural History Museum, Bergen Museum, University of BergenBergen, Norway

**Keywords:** Alien species, cryptic species, tropical ecology, biodiversity, ecological genetics, fronzen niche variation model, general-purpose genotype model, Scolytinae

## Abstract

The wide distribution and dominance of invasive inbreeding species in many forest ecosystems seems paradoxical in face of their limited genetic variation. Successful establishment of invasive species in new areas is nevertheless facilitated by clonal reproduction: parthenogenesis, regular self-fertilization, and regular inbreeding. The success of clonal lineages in variable environments has been explained by two models, the frozen niche variation (FNV) model and the general-purpose genotype (GPG) model. We tested these models on a widely distributed forest pest that has been recently established in Costa Rica—the sibling-mating ambrosia beetle *Xylosandrus morigerus*. Two deeply diverged mitochondrial haplotypes coexist at multiple sites in Costa Rica. We find that these two haplotypes do not differ in their associations with ecological factors. Overall the two haplotypes showed complete overlap in their resource utilization; both genotypes have broad niches, supporting the GPG model. Thus, probable or not, our findings suggest that *X. morigerus* is a true ecological generalist. Clonal aspects of reproduction coupled with broad niches are doubtless important factors in the successful colonization of new habitats in distant regions.

## Introduction

Few organisms regularly succeed at colonizing and establishing new populations in distant regions. In plants and small invertebrates, the mode of reproduction plays a crucial role in colonization and extreme inbreeding is a common feature of successful taxa. Plants in peripheral, isolated, and island habitats are commonly characterized by self-compatible mating systems (Baker 1955; Herlihy and Eckert 2005). Indeed, the transition from outcrossing to selfing is one of the commonest evolutionary trends in plants (Stebbins 1974), presumably due to the advantages of reproductive assurance (Herlihy and Eckert 2002). Similarly, regular sib-mating, oedipal mating, and parthenogenesis are overrepresented among successfully colonizing species of small invertebrates (e.g., Simberloff 1986; Adamson and Ludwig 1993; Haack 2001). The reason why these alternative mating systems are advantageous for colonization is clear: sibling mating before dispersal, or mother–son mating, selfing, or parthenogenesis after dispersal, eliminate the many difficulties associated with mate finding in animals and with pollination in monoecious plants. Colonizing species with a previous history of inbreeding, and apomictic species, have a further advantage over obligate outbreeders: they suffer little or no inbreeding depression as a result of (previous) purging of deleterious recessive alleles (Husband and Schemske 1996; Haag and Ebert 2004; Peer and Taborsky 2005).

Inveterate inbreeding should quickly result in largely homozygous genotypes that reproduce as quasi-clonal family lineages (with the exception of polyploid selfing plants, e.g., Brochman et al. 2004). Advantageous multilocus genotypes will be faithfully reproduced from one generation to the next, maintaining favored coadapted gene complexes. At the same time, background selection, selective sweeps, genetic drift, and repeated colonizations and extinctions will all reduce within-population variation (Hartl 1971; Nur 1971; Selander and Hudson 1976; Charlesworth 2003), leading to homogeneous or genetically depauperate small populations. Parallels can therefore be drawn between inbreeding and parthenogenetic reproduction (Lynch 1984; Hamilton 1983; Peer and Taborsky 2005), and regular inbreeding can be considered a form of clonal (technically, pseudoclonal) reproduction.

Low levels of within- and between-individual genetic variability should restrict the ability of dispersing inbreeders to adapt to new conditions encountered after dispersing to new sites (White 1973; Lee 2002), but inbreeding plants and animals are often good long-distance colonizers and many are widely, even globally, distributed. Contributing to their colonizing success is the fact that regularly inbreeding plants and animals have relatively broad niches and large ranges (Baker 1955; Kirkendall 1993; Zangerl and Bazzaz 1984; Randle et al. 2009; but see Loxdale et al. 2011). Two hypotheses have emerged in recent years that treat the evolution of niche breadth in clonally reproducing organisms. Though originally developed in the context of competition between sexual and asexual populations, they are equally applicable to understanding the ecology and evolutionary stability of regular inbreeding. The two theories differ in the ecological niche breadth of asexuals, though the approaches are not mutually exclusive; both can be applied to understanding geographic distribution patterns (including, by extension, colonizing success) of clonally reproducing forms (Vrijenhoek and Parker 2009). The frozen niche variation model (FNV: Vrijenhoek 1979, 1984, 1998) posits that individual clones are relatively narrowly adapted and that, after interclonal competition, different clones occupy different ecological niches. This model supposes that multiple asexual clones have arisen from sexuals, each “freezing” a portion of the overall ecological niche space of the ancestral sexual population. The model was developed initially to explain the maintenance of sex in populations invaded by multiple clones, but is also a model for clonal coexistence. The general-purpose genotype model (GPG; Baker 1965) treats clones as ecological generalists. This assumption has a long pedigree: for over 70 years, ecologists have observed that the colonizing success of parthenogenetic or self-fertilizing species is regularly associated with genotypes with exceptionally broad ecological tolerances (e.g., Vandel 1928; Baker 1965; White 1973, Jaenike et al. 1980; Lynch 1984; Vrijenhoek and Parker 2009; but see Loxdale et al. 2011). The broad ecological niches of generalist clones increase the probability that a species can invade a new region, and this is believed to be one of the main reasons that parthenogenetic species are such successful invaders (Baker 1965; Parker et al. 1977). The GPG model predicts that over time in a heterogeneous environment, clones with the lowest geometric mean fitness will go extinct leaving only the more successful broadly generalized genotypes with higher geometric mean fitness (Weeks and Hoffmann 1998).

Although consistent close inbreeding is said to be rare in nature (Ralls et al. 1986; Thornhill 1993), this reflects a view biased toward vertebrates; regular inbreeding occurs in a wide variety of invertebrate groups (Ghiselin 1969; Jarne and Charlesworth 1993; Wrensch and Ebbert 1993). Regular inbreeding has been particularly successful both ecologically and in an evolutionary context in the weevil subfamily Scolytinae (bark beetles), with about 1500 species resulting from at least eight different origins of brother–sister mating (Kirkendall 1993; Farrell et al. 2001). About one-fourth of all species of Scolytinae are inbreeders; roughly half of all tropical species, and four of five species on tropical islands, inbreed (Kirkendall 1993; Jordal et al. 2001). Many of these species are widely distributed and abundant; host specialization is the exception rather than the rule, as most species have been collected from multiple plant families (Wood and Bright 1992). In the largest clade of regularly inbreeding beetles (Xyleborini and *Coccotrypes*), all species reproduce by brother–sister mating and are haplodiploid (Normark et al. 1999).

Does the striking success of globally distributed inbreeding Scolytinae species result from generalist genotypes, or do populations of these beetles comprise a variety of genotypes specialized to hosts, host tissue, or other key niche variables? As a first attempt at addressing this key question, we test here these two hypotheses in one of the most ubiquitous and abundant inbreeding species of Scolytinae, *Xylosandrus morigerus* (Blandford). This tiny wood-boring beetle is native to the tropical regions of the Indian subcontinent, Southeast Asia, and Papua New Guinea, but has recently become established in all tropical regions of the world, including many isolated oceanic islands (Anonymous 1971; Jordal et al. 2001).

Three important features of *X. morigerus* and its relatives facilitate the successful establishment of new demes (Kirkendall and Ødegaard 2007). First, mating normally occurs between siblings and before dispersal, which assures successful insemination of most dispersing females. Second, being haplodiploid, females that are not inseminated by a brother before leaving the nest can potentially mate with a haploid son produced from unfertilized eggs (Herfs 1959; Norris 1992; but see Biedermann 2010). A single female is therefore sufficient to start a new population distant to its area of origin. A third contributing factor is the extremely broad range of host plants that these beetles can utilize for breeding and feeding (Beaver 2005), facilitated by a tight symbiosis with ambrosia fungi. *Xylosandrus* species are mainly associated with one or several species of *Ambrosiella* fungi and occasionally other imperfect ascomycete fungi (Batra 1966). Fungal spores are borne in a highly specialized and invaginated, glandular cuticular structure in the mesonotum (mycangium) and inoculated into the wood via the tunnel wall. The ambrosia fungi can grow in a taxonomically broad range of host trees, and in a wide variety of host plant tissues, including wood or pith of small branches, twigs, and woody leafstalks (Kalshoven 1958, 1961, 1963; Browne 1961; Beaver 1976); given its catholic habits, *X. morigerus* can readily colonize a tropical landscape with a species composition quite different from that of the beetles’ source population.

The earliest Neotropical records of *X. morigerus* are from Brazil in 1940 (Beaver 2005) and Colombia in 1959 (Wood 2007). The species is now widespread and abundant in the region suggesting a rapid, recent geographical expansion, though perhaps coupled with multiple new introductions. Preliminary DNA sequencing of 28 Costa Rican specimens revealed only two, highly divergent cytochrome oxidase I (COI) haplotypes. The two COI lineages co-occur in many localities in Costa Rica, where *X. morigerus* is among the most abundant scolytine species in wet lowland forests (Wood et al. 1991; Kirkendall, unpubl. data). This situation provides an interesting test of competing hypotheses of niche breadth for explaining the successful establishment of inbreeding ambrosia beetles: is the recent colonizing success of the neotropics by *X. morigerus* due to general purpose genotypes, or to a mixture of well-adapted more specialized ones?

Our GPG-based hypothesis was that there would be no association between genotype and our environmental variables. In this case, we should observe similar fitness for the two divergent genotypes when in the same resource. The alternative, FNV-based hypothesis was that genotypes would prove to be ecologically different (more specialized), with different resource preferences and unequal fitness when occurring in the same resource. In an attempt to detect genotype-specific resource specialization, we measured reproductive success of the two haplotypes in very different resource units, fallen *Castilla* branches and fallen *Cecropia* leaves, in differing tissues (pith vs. cortex or bark), and in different microhabitats (resource unit hanging, partially suspended, completely on the ground).

We are assuming that differences among females in host-choice behavior or in host- or host tissue-related fitness would reflect underlying genetic differences. In effect, we are using a fragment of COI, a mitochondrial gene, as a marker for the genome as a whole. We are assuming, then, that genomes with COI sequences that are highly divergent will also differ in nuclear genes such as those that affect behavior and ecology. Linkage between COI and nuclear genes is expected to be strong in regularly inbreeding species, as a result of long-term inbreeding with no or very little outcrossing (Charlesworth 2003). With each generation of sib-mating, homozygosity increases and hence recombination is reduced (Narain 1966). The expected and observed result of this effect plus the effects of selective sweeps, background selection, and genetic bottlenecks is that genetic diversity within populations is severely reduced in inbreeders, at the same time that protein evolution is accelerated, and thus coexisting inbred lineages are expected to become highly divergent (Charlesworth and Wright 2001; Graustein et al. 2002; Charlesworth 2003). As a first attempt at checking if such linkage does occur in *X. morigerus*, we tested for a significant association between the mitochondrial COI gene and the two nuclear loci carbamoyl phosphate synthetase 2 aspartate transcarbamylase dihydroorotase (CAD) and 28S.

We report here a high divergence in Costa Rican mtDNA sequences for *X. morigerus*—nearly 10%. Loxdale et al. (2011), in a recent, provocative paper, claim that insects traditionally considered to be ecological generalists frequently turn out to harbor cryptic species, going as far as to label ecological generalism “improbable.” We therefore carefully studied the collected material for morphological differences, and we applied the nuclear DNA results to assess this possibility.

## Materials and Methods

### Sample collection

Data were collected at La Selva Biological Station (10°26′N, 83°59′W) in Costa Rica between 20 June and 11 July 2006. We collected beetles from fallen branches of the Panamanian rubber tree *Castilla elastica* (Moraceae) and from petioles of fallen leaves of *Cecropia* (Urticaceae). These were by far the most abundant resource units for *X. morigerus* at La Selva, which species has otherwise been collected from a wide variety of branches, vines, fallen leaves, and fallen fabaceous pods at the reserve and elsewhere in the country (L. R. Kirkendall, unpubl. data). *Castilla* branches and *Cecropia* leaves fall year round and are readily available throughout the forests of La Selva. These trees are native to the Neotropics and very common in disturbed forest patches such as tree falls and riverbanks.

A total of 257 colonized branches and leaves were collected and dissected. Ecological data recorded included host species, plant tissue, forest type, temperature and moisture content, the position of the fallen resource relative to the ground, and light exposure. During dissection of the branches and leafstalks, we recorded the presence or absence of ambrosia fungi, indicative of successfully established nests, and the combined number of adults, pupae, larvae, and eggs, as a quantitative measure of reproductive success.

The surface temperature of each resource unit (branch or leaf) was recorded to the nearest whole-degree °C using an infrared thermometer (Fluke 62 Mini Infrared Thermometer Gun: Fluke, Everett, WA, USA). Moisture content was measured in the field using a timber moisture meter (Pin-type model MT909: Electrophysics, Dutton, Ontario, Canada). To control for meter inaccuracy, we dried the same branches and leafstalks for 1–2 days at 60°C until the difference between the last weightings was <1%. The moisture percentage was then calculated using initial weight and oven-dried weight loss (M = ((W_wet_– W_dry_)/W_wet_) × 100). We used three categories for the light conditions experienced by each branch or leaf: fully shaded (>2/3 shade), partly shade (>1/3 shade, <2/3), and full light (<1/3 shade). The degree of shade was determined visually by estimating the amount of sunlight reaching the ground at the area containing the branch or leafstalk. The position relative to the ground was also noted and divided into three categories: hanging from vegetation, with no contact with the soil or leaf litter; partly on the ground, partly suspended; full contact with the ground, where minimum two-thirds of the resource unit was in direct contact with the soil or leaf litter. For *C. elastica*, there were no examples of hanging branches, because these branches are heavier and rarely trapped by understory vegetation.

### Assignment of COI haplotypes

DNA extraction was performed in 150 µl InstaGene™ Matrix (Bio Rad Laboratories, Hercules, CA, USA) using tissues from whole individuals. Only one specimen per family group was included. A 1299 base pair long fragment of the mitochondrial gene COI and the 5′ end of the tRNA_leu_ was amplified by the polymerase chain reaction (PCR) using primers S1718, (5′-GGA GGA TTT GGA AAT TGA TTA GTT CC-3′) and A3018, (5′-TCC AAT GCA CTA ATC TGC CAT ATT A-3′) (Simon et al. 1994). The PCR reaction contained 14.3 µl ddH_2_O, 2.5 µl 10× PCR Reaction Buffer II, 2.0 µl MgCl_2_ (25 mM), 2.0 µl dNTPs, 0.2 µl AmpliTaq® DNA Polymerase (Applied BioSystems, Foster City, CA, USA), 1.0 µl each forward and reverse primer (10 µM), and 2.0 µl of DNA, for a total of 25 µl mixture. Amplification cycles consisted of one initial denaturation step at 94°C for 2 min, followed by 35 cycles of denaturation for 30 s, annealing at 50°C for 1 min, and primer extension at 72°C for 1 min. A final extension step included 7 min at 72°C. PCR products were purified by digesting excess nucleotides by EXOSAP-IT® (USB). We sequenced 47 PCR products in both directions using standard cycling conditions (Applied BioSystems, Foster City, CA, USA).

**Figure 1:**
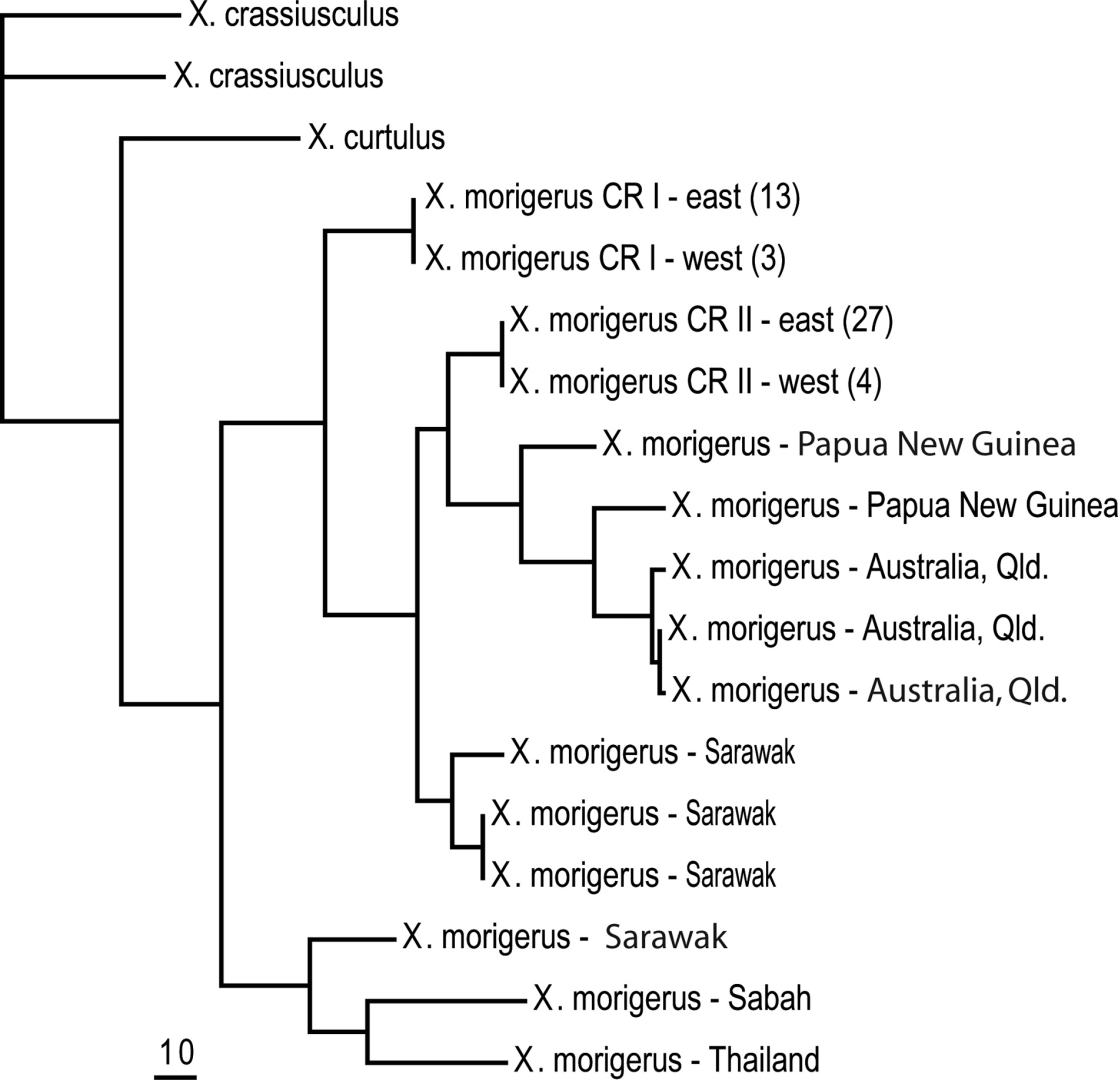
Phylogeny of *Xylosandrus morigerus* populations based on maximum parsimony analysis of COI sequences done in PAUP (hs, addseq = random, nrep = 200). The topology was rooted with *X. curtulus* and *X. crassiusculus*. Number of identical haplotypes is given, for cases with shared haplotypes. Localities in western Costa Rica include Golfito and San Vito, Puntarenas province; in eastern Costa Rica, all samples are from La Selva Biological Research Station, near Puerto Viejo de Sarapiquí (Heredia province).

Only two haplotypes had been detected from Costa Rica (see Fig. 1), both confirmed for the La Selva study area by sequencing the current PCR products, and here designated CR I (*N* = 16) and CR II (*N* = 31). With as much as 9.1% divergence at the nucleotide level, we could readily develop a PCR-RFLP (Restriction Fragment Length Polymorphism) protocol that discriminated between the two haplotypes. Using Sequencher™ 4.5 to find cutting sites, we chose the restriction endonuclease Nla IV (= Bsp LI). Nla IV cuts CR I at two separate sites, between 417 and 418 and between 686 and 687, resulting in three bands with base pair lengths of 269, 417, and 521. The other haplotype, CR II was cut at one site by Nla IV, between 592 and 593, resulting in two bands with base pair lengths of 592 and 690. The enzyme mixture consisted of 0.1 µl 10mg/mL BSA (Bovine Serum Albumin), 9.0 µl 2× NEB 4 reaction buffer (New England BioLabs® Inc., Ipswitch, MA, USA), 1.0 µl Nla IV (New England BioLabs® Inc., Ipswitch, MA, USA), and 10.0 µl PCR product to a total of 20.0 µl mixture digested in 37°C for a minimum of 1 h. Cut products were visualized on a 1.5% agarose gel.

Two nuclear genes were sequenced to confirm the species status of the two deeply diverged mitochondrial haplotype, and to test for a significant association between mitochondrial nuclear loci. Ten specimens—five specimens of each COI haplotype—were amplified for fragments of the large ribosomal subunit 28S using the primers S3690 and A4394 and of CAD using the primers CADfor4 and CADrev1mod (see Jordal et al. 2011). PCR cycling conditions followed those for COI, with annealing temperature 55°C for 28S. DNA sequences are deposited in GenBank under the following accession numbers JN982488-JN982498 (COI), JN982499 (28S), and JN982486-JN982487 (CAD).

### Morphology

Given the large divergence at COI (almost 10%), it behoved us to examine the two haplotypes for morphological differences that could indicate the existence of cryptic species. Three of us (BJ, MK, and LRK) independently examined females of known haplotype, taken from different families and different localities.

### Statistical methods

All statistical analyses were executed in the software packages R Gui 2.4.1 and Microsoft Excel (2007 Professional). A Generalized linear model (GLM) was designed for linear regression analyses using forward selection on AIC (Akaikes's Information Criterion = –2 × log – likelihood + 2*p*, where *p* is the number of parameters) to determine the ecological variables (host type, breeding tissue, diameter, ground position, shade, and moisture) possibly affecting the two haplotypes. To test for the best fit of models, an analysis of variance (ANOVA) with a χ^2^ test was used in conjunction with the binomial distribution used in the GLM. In addition, χ^2^ tests (Pearson) on contingency tables were used to test for interactions between haplotypes and ecological variables. The contingency tables had a larger sample size than the model due to the fact that a model requires a dataset without missing values. We, furthermore, examined whether a certain haplotype was more successful in establishing a breeding chamber and producing offspring using two different approaches. In the first approach, we fitted a GLM to the data with the binomial response variable of brood present or absent (1/0). The second approach used brood size as the response variable, excluding failed or fresh chambers without any offspring. Due to high overdispersion in the brood size data, the distribution type was changed from Poisson to quasi-Poisson.

## Results

### Linkage to nuclear genome

Four individuals of each COI haplotype were successfully sequenced for COI, 28S, and CAD. For the 692-bp fragment of COI, CR I and CR II differed by 9.5%. There was no intraspecific variation in 28S. Two CAD haplotypes were found, which differed by 1.1% (460 bp). There was complete concordance between nuclear (CAD) and mitochondrial (COI) DNA variation: CR I individuals had one CAD haplotype, CRII had the other (Fisher's exact test, *P* = 0.03).

### One species or two?

Despite careful independent examination by three of us of all external features known to differ between closely related species of xyleborine ambrosia beetles, no consistent differences could be found between the two COI + CAD types. The lack of variation at 28S also argues for there being only one species in the material from Costa Rica: this gene does vary among species of xyleborines, and typically varies little or not at all within species (Dole et al. 2010).

### Haplotype distribution

In the 257 *Castilla* branches and *Cecropia* leafstalks examined, 785 families were found, with a maximum of 36 families in a single branch. We succeeded in amplifying DNA from representatives of 625 of the 785 families; 38% of families were haplotype CR I and 62% haplotype CR II.

A General linear model (GLM) was fitted to the ecological variables host plant, host tissue, ground position, moisture, shade, and host diameter, to test for association with each of the two haplotypes (Table 1). Host plant (*Castilla* branch or *Cecropia* leafstalk) and host tissue (pith, cortex/bark) were the only two variables with a significant effect on the distribution of haplotypes (*P* < 0.01, 0.03, *N* = 441, rows containing missing data removed). Both variables were significant separately, but the model containing their interaction explained better the haplotype distribution.

**Table 1:** GLM assessing which ecological variables predict the distribution of haplotypes (ANOVA, family = binomial, test = chi square). P values with asterix (*) in this and following tables are significant at <0.05

Response	Predictors	*P* (>|chi|)	Interaction	*P* (>|chi|)
Haplotype	+Host type	0.01*	Host type × Breeding tissue	0.02*
	+Breeding tissue	0.03*	Host type × breeding tissue × ground position	0.22
	+Ground position	0.07		
	+Shade	0.09		
	+Poly (Diameter, 2)	0.12		
	+Moisture	0.45		

**Figure 2:**
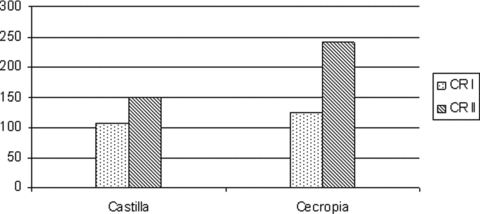
Distribution of haplotypes CR I and CR II with respect to the host plants *Castilla* (Moraceae) and *Cecropia* (Urticaceae).

**Figure 3:**
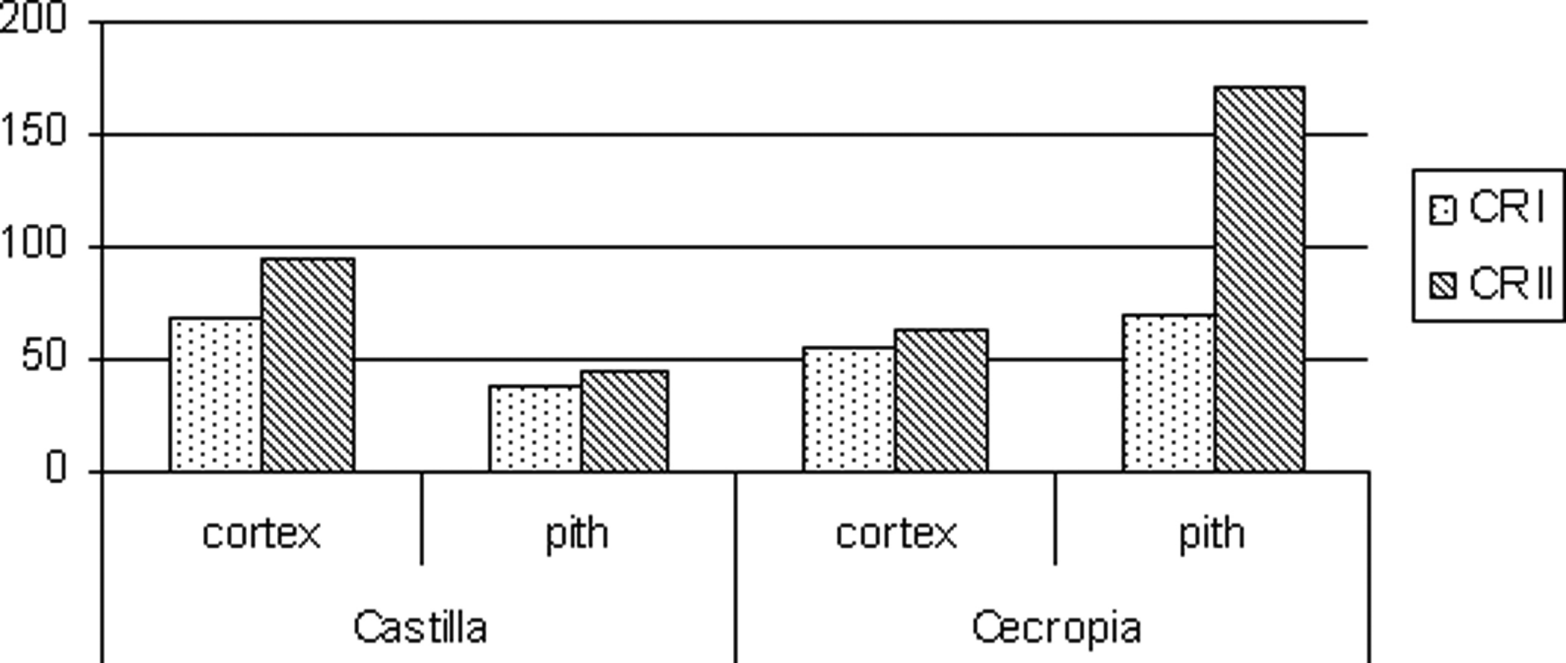
Distribution of haplotypes CR I and CR II with respect to host plant tissue type (cortex vs. pith) for the host plants *Castilla* and *Cecropia*.

**Figure 4:**
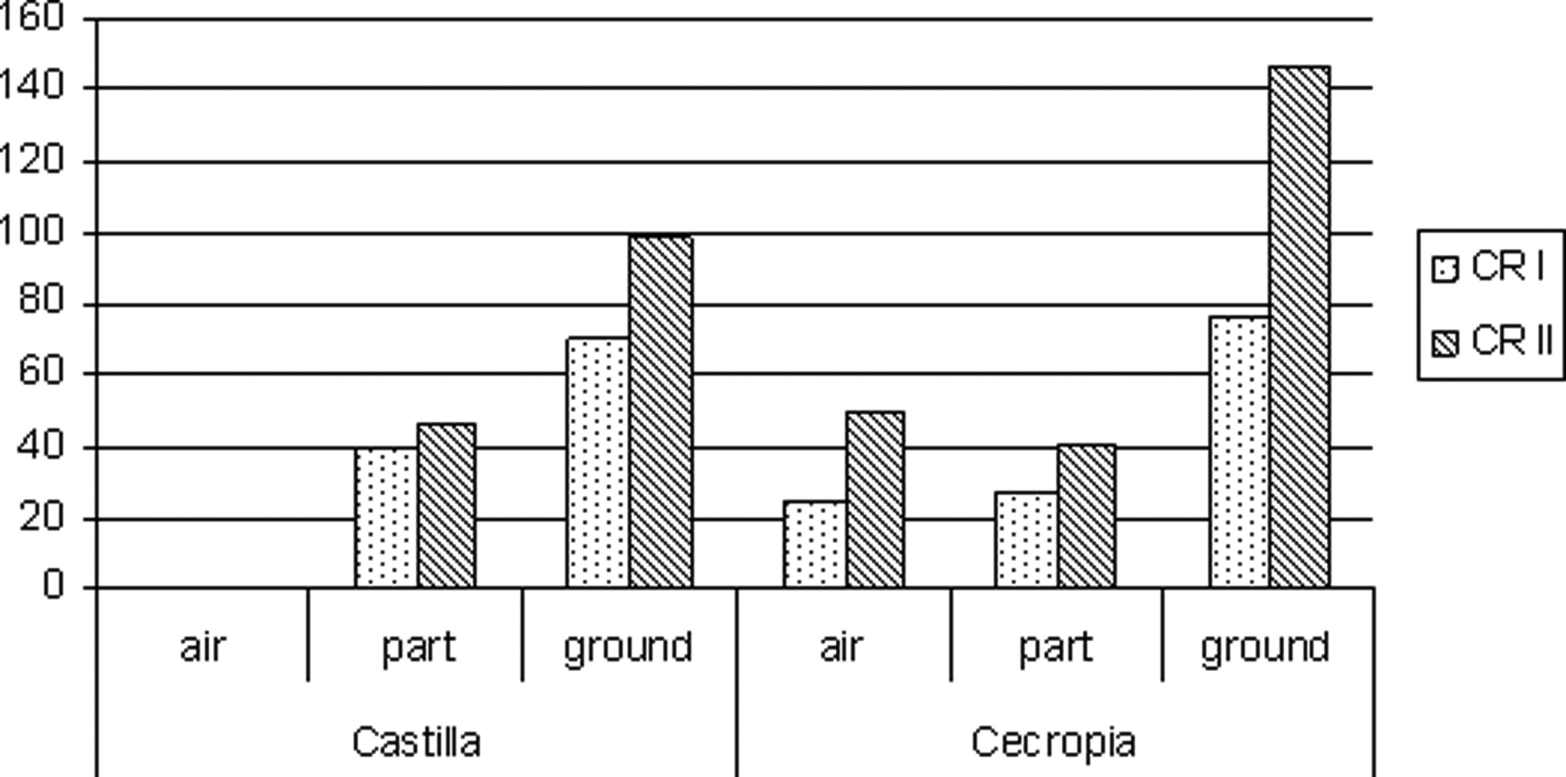
Distribution of haplotypes CR I and CR II with respect to host plant and the position of leafstalks and branches (air, hanging on vegetation; part, in partial contact with ground; ground, full contact with ground).

**Figure 5:**
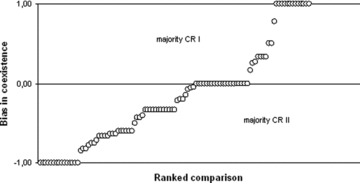
Coexistence of reproducing females with haplotypes CR I or CR II. Bias index varies from +1, all individuals with haplotype COI, to −1, all with COII.

To confirm the results from the GLM, additional contingency tables were analyzed using χ^2^ tests for each variable. There was a significant association between host plant (*Castilla* branches or *Cecropia* leafstalks) and haplotype (χ^2^ test, *P* < 0.04). CR II was found more often in leafstalks than in branches, while CR I showed no such preference (Fig. 2). The overlap in host plant use was nevertheless considerable and both haplotypes frequently co-occurred in the same resource unit (Fig. 3). There was, furthermore, a significant association between the haplotypes and host tissue (C = cortex/bark, P = pith) used for breeding (χ^2^ test, *P* < 0.01), mainly due to variation within leafstalks (χ^2^ test, *P* < 0.01, *N* = 359) but not in branches (*P* = 0.65, *N* = 244). CR II was much more frequently collected from pith of leafstalks than was CR I (Fig. 4). The remaining variables were not associated with haplotype, including “ground position” that was nearly significant in the GLM analysis (Table 1). However, because host plant was a significant variable in explaining haplotype distribution, variation in ground position was further analyzed for each host plant separately showing a significant difference between the two haplotypes for leafstalk position (*P* < 0.01, *N* = 362) but not for branch position (*P* = 0.48, *N* = 258). The difference observed for leafstalks was possibly due to the higher frequencies of CR II in leafstalks with ground contact (Fig. 5).

### Variation in fitness

The simplest GLM showed no significant effect of haplotype on the success of breeding (i.e., brood present or absent, ANOVA χ^2^ test, *P* = 0.72) (Table 2). Further modeling was conducted to identify other variables that may have an effect on reproductive success, resulting in a significant association between breeding chamber establishment and host type (*P* < 0.01), and host diameter (*P* < 0.01).

**Table 2:** GLM assessing which ecological variables predict if females successfully establish broods (ANOVA, family = binomial, test = chi square)

Response	Predictors	*P* (>|Chi|)
If offspring	Haplotype	0.72
	+Host type	0.01*
	+ Breeding tissue	0.16
	+ Ground position	0.45
	+Poly (Diameter, 2)	0.01*
	+Moisture	0.80
	+Shade	0.08

**Figure 6:**
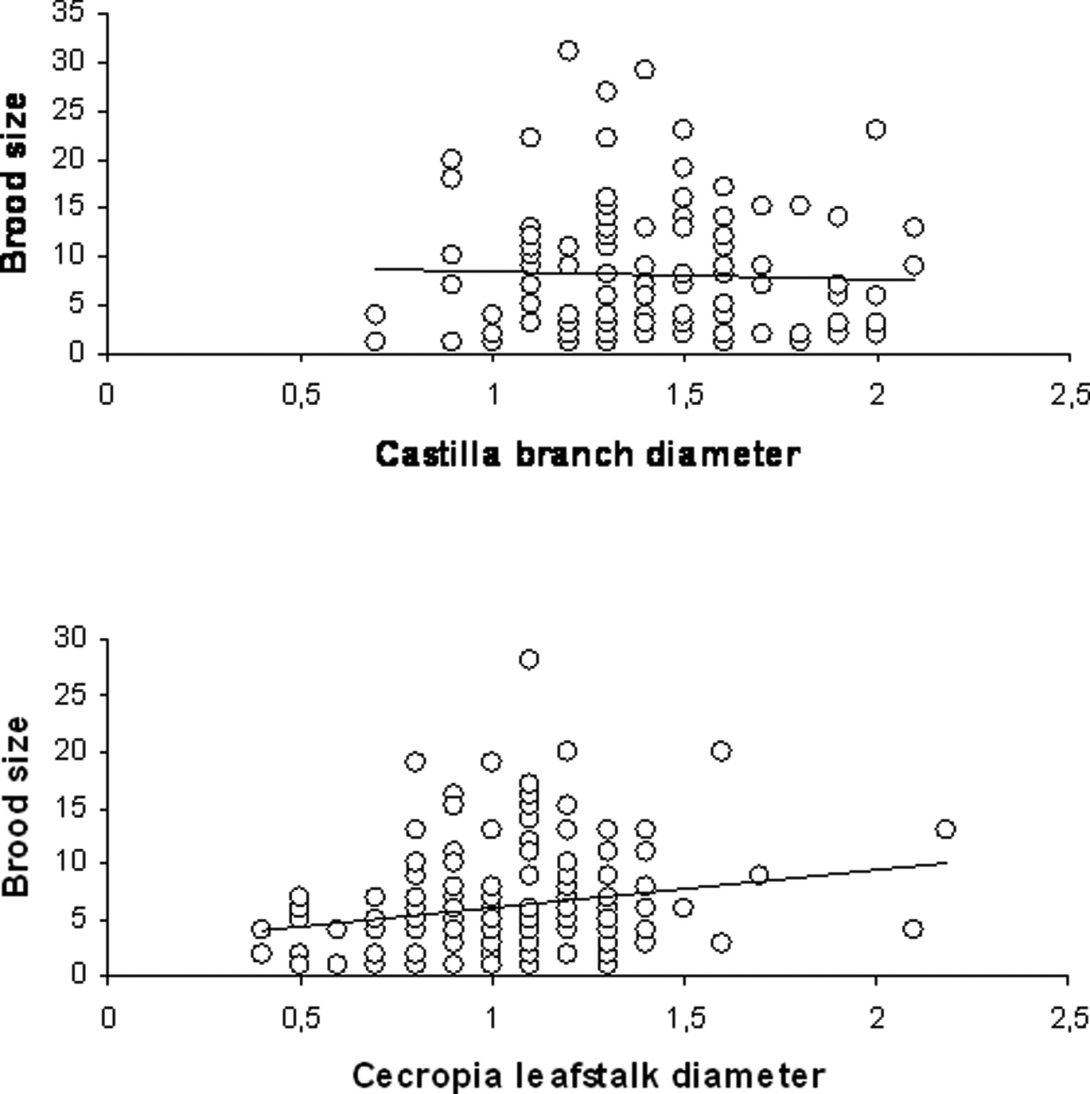
Distribution of brood size with respect to host-plant diameter for *Castilla* (above) and *Cecropia* (below).

**Figure 7:**
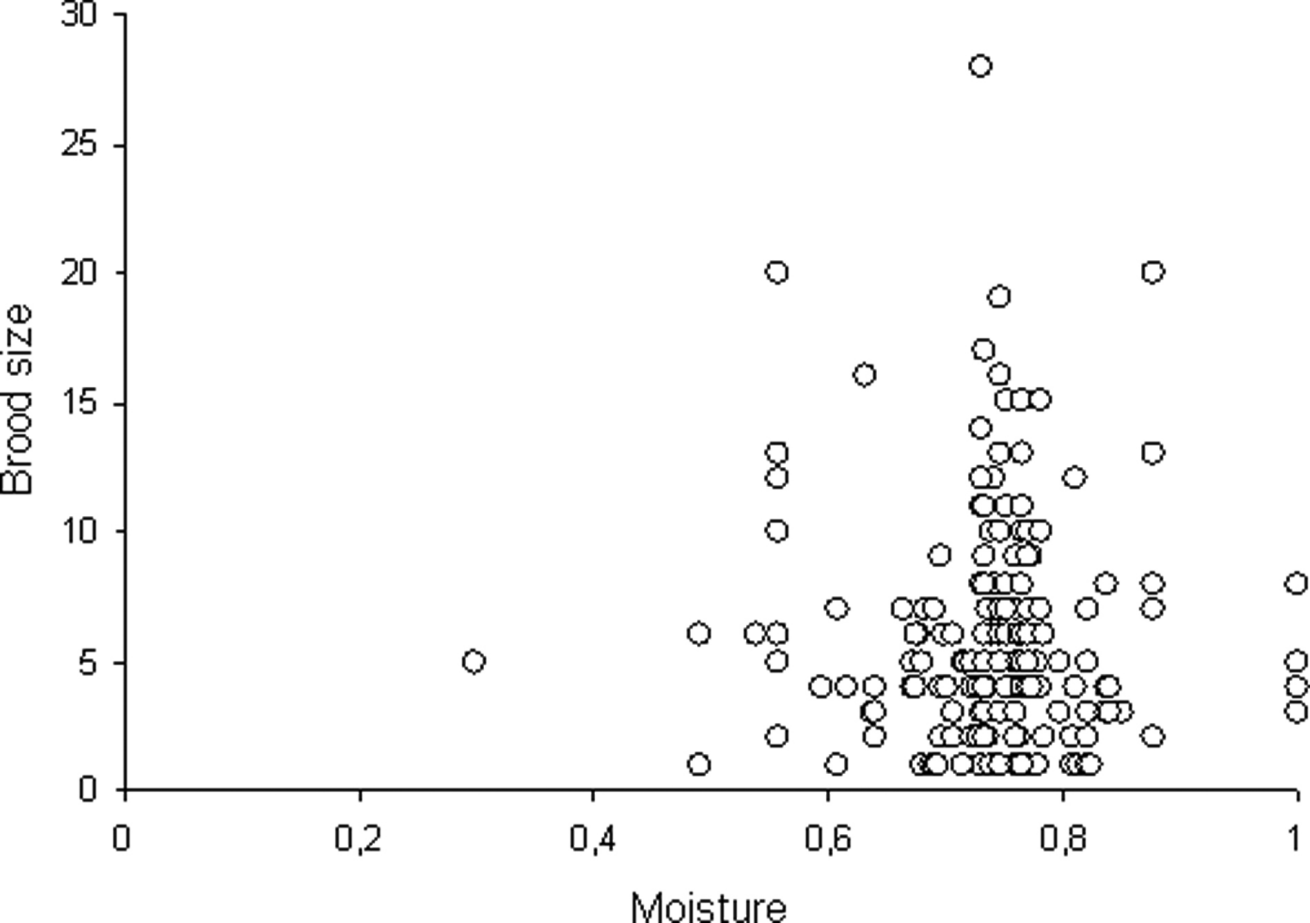
Distribution of brood size with respect to host-plant moisture content.

The average brood size for successfully established families was 7.1 (*N* = 381), ranging from 1 to a maximum of 32 (Table 3). Based on a GLM model fitted to the same data with total offspring as the response variable (quasi-Poisson), haplotype had no significant effect on brood size (Table 4: ANOVA, test = F, *P* = 0.91). Three other factors had a significant impact on brood size: host type, *P* < 0.01; breeding tissue, *P* < 0.02; and ground position, *P* < 0.02. Brood sizes were on average higher in *Castilla* branches (pith) compared to *Cecropia* leafstalks (cortex tissue), respectively, and slightly higher in host material on the ground (data not shown). The highest brood sizes were furthermore found in branches and leafstalks with medium to largest diameter, particularly so for *Castilla* branches (Fig. 6), and in breeding tissue with 60–85% moisture (Fig. 7).

**Table 3:** Average brood size produced by females with different haplotypes in different host plants

Host	*Castilla*		*Cecropia*	
		
Haplotype	CR I	CR II	CR I	CR II
Average	7.80	8.47	6.31	6.24
Max	32	29	28	20
Sample size	*n* = 44	*n* = 71	*n* = 68	*n* = 112

**Table 4:** GLM assessing the effect of ecological variables on brood size (ANOVA, family = quasi-Poisson, test = F)

Response	Predictors	Pr (>F)
Total Offspring	Haplotype	0.91
	+Host type	0.00*
	+Ground position	0.01*
	+Breeding tissue	0.01*
	+Poly (Diameter, 2)	0.08
	+Shade	0.79
	+Moisture	0.88

## Discussion

### The GPG and successful invasions

The similarly broad resource usage and considerable overlap in ecological parameters between highly differentiated genetic lineages clearly demonstrated that individuals of *X. morigerus* are truly generalists, corresponding to the GPG model. Although we did find a small, but significant, bias in the distribution of CR II haplotypes with respect to breeding tissue (pith vs. cortex) in *Cecropia*, the overall pattern for the two haplotypes combined revealed very little differentiation in ecological preferences. This is perhaps best illustrated by the co-occurrence of both haplotypes in 64 of those 101 cases where multiple colonization's occurred in the same resource unit. The relatively higher occurrence of CR II in *Cecropia* might indicate a slight degree of ecological differentiation between haplotypes. However, any selective advantage of the higher breeding frequency in *Cecropia* by CR II is counterbalanced by lower brood production in this host plant and cryptic specialization as predicted by the FNV model is therefore quite unlikely.

GPGs were originally proposed to explain the success of invasive weedy plants that show broad adaptations to different growth conditions (Baker 1965). Recent studies of a variety of organisms have found low to nonexistent differentiation among clones along ecological gradients (e.g., Selander and Hudson 1976; Jaenike et al. 1980; Jensen et al. 2002; VanDyke et al. 2004), as we found with the minimal differences between the two *X. morigerus*“pseudoclones” in this study. The GPG model relates the invasive success of clonal species to their broad tolerance of environmental variation, which makes them less vulnerable to novel environments in new areas despite a lack of genetic variation. This makes intuitive sense; a successful invasive species will have a higher probability to find a suitable ecological niche if it is a broad-spectrum generalist (e.g., Baker 1965, Parker et al. 1977). Once a successful generalist haplotype has become established in a region, it is likely that it will remain so for a long time.

### Is genetic variation important?

The presence of only two different haplotypes throughout Costa Rica and Panama for the recently established, but yet highly abundant *X. morigerus*, demonstrates how populations with low to nonexistent genetic variation are capable of coping with a range of environmental variables and growing to an enormous population size. Under this scenario it appears that genetic variation is not a critical factor, perhaps as a result of GPGs in these beetles. Although genetic variation *per se* could be underestimated by using a single mitochondrial marker, the genetic variation at nuclear and mitochondrial genes has been shown to be similar in regularly inbreeding species because recombination is effectively absent in such species (Birky et al. 1983; Graustein et al. 2002). It is therefore not expected to find finely divided genetic variation at most nuclear genes in inbreeding species such as *X. morigerus,* where outbreeding is extremely rare.

We have mentioned that GPGs allow considerable plasticity in ecological parameters at the individual level as one way to cope with environmental variation. However, there are other means for maintaining or recreating also genetically founded variability, mainly through the mixing of genotypes by long-distance immigration. In the case of *X. morigerus*, two independent colonizations of Costa Rica has taken place during the last few decennia and clearly shows that long-distance migration is sufficiently frequent and thus a likely factor in maintaining local genetic variation. It is of course not certain that both or any of these genetic lineages will survive in Costa Rica in the long run, but our ecological data on host-plant associations do not indicate that selection is sufficiently strong to exclude one of these pseudoclones. Although the GPG model predicts that a population will become monoclonal after periods of interclonal competition (Parker 1979; Lynch 1984; Vrijenhoek and Pfeiler 1997), multiple clones may coexist over longer time scales if fitness varies over time (e.g., Jaenike et al. 1980; Lynch 1984; Weeks and Hoffman 1998). This aspect of nonequilibrium theory was originally proposed to explain coexistence of species with identical niches, but can be extrapolated to account for clones (see e.g., Chesson 1986). To enable a more definite answer to the question about survival of coexisting genetic lineages, measurements of pseudoclone performance would have to be measured over a much longer time than was available for our study.

### Resource quality of the hosts

The average brood size was well below 10 in both *Castilla* branches and *Cecropia* leafstalks, which is lower than reported from some other host species. Brood size data therefore indicate that branches and leafstalks of our study plants are poorer habitats than many native Asian host plants (Kalshoven 1961). The maximum brood size measured in our study (32) is nevertheless not very much lower than the highest reported from other hosts such as *Coffea robusta* (maximum brood size of 70: Kalshoven 1961), and Beaver (1976) reported an unusually low mean mature brood size of 6 (*N* > 100 broods) for this species taken from a variety of hosts on Samoa. Specialist bark beetles in *Cecropia* leafstalks (such as species of *Scolytodes*) do not produce any larger broods in this host material (see Jordal and Kirkendall 1998). We also note that growth conditions for the ambrosia fungi on which they feed are sufficient for successful brood production. Moisture content in the host material is an essential factor for successful breeding in ambrosia beetles, which is likely coupled with specific growth requirements of their symbiotic fungi (Nobuchi 1972). Typical values for ambrosia beetles average around 60–85%, as typically measured in successfully breeding *X. morigerus* (Fig. 6).

### Are lineages clone-like?

That *X. morigerus* lineages are effectively clonal remains to be rigorously tested, though it is at least supported by our preliminary linkage results. Future studies will focus on measuring outbreeding rates in this and similar inbreeding bark beetle species. The few studies that have quantified inbreeding in sib-mating scolytines have reported extremely high values for *F*_IS_ (the inbreeding coefficient), values ranging from 0.5–0.9 (Holzman et al. 2009; Gauthier 2010). Though direct data are lacking for *X. morigerus*, a variety of indications suggest that mating between females and unrelated males (exogamy) is rare. We have not observed males of *X. morigerus* outside their galleries, during this study or other field work, and in this study only one case of merging galleries was observed among the 783 galleries dissected. Neither has evidence of outbreeding been reported previously for this species (e.g., Speyer 1923; Kalshoven 1958, 1961, 1963; Browne 1961; Wood 1982; Wood and Bright 1992), though wandering males have been observed for the related temperate species *X. germanus* that, it should be noted, breeds in rather dense colonies on tree trunks (Peer and Taborsky 2004). More generally, *X. morigerus* typifies the extreme inbreeding syndrome described in W. D. Hamilton's (1967) landmark paper “Extraordinary sex ratios.” As described for many species where outbreeding is rare, males are dwarfed, flightless, blind or nearly so, and rare in number—characteristics which are only adaptive when males seldom compete with nonbrothers for matings. In *X. morigerus* and related species, mothers usually produce just one male, the minimum necessary for fertilizing all their daughters (Kirkendall 1993; this study, unpubl. data). Finally, we would point out the possibility that local populations of extreme inbreeders will likely consist of one or a few lineages; a considerable frequency of the few nonsibling matings, which occur, will be between males and females of the same lineage. Taken together, then, the behavior and morphology of *X. morigerus* and more general considerations suggest that lineages may be reproducing clonally for long periods of time.

## Conclusion

This study is the first to examine the interaction between environment and genotypes in a highly inbred scolytine beetle. In addition to providing insight into the underlying reasons for success in inbred invasive species, studying a system that can be thought of as pseudoclonal can contribute to the long and still ongoing debate regarding the advantages of sex and outbreeding (e.g., Maynard Smith 1978; Bell 1982; Lynch 1984; Hurst and Peck 1996; Vrijenhoek 1998; West et al. 1999; Lundmark and Saura 2006; Vrijenhoek and Parker 2009). A generalist genotype combined with the ability to start a new population based on a single female provides a powerful combination for successfully invading new areas, and we believe that this must have played a central factor in the recent global expansion of *X. morigerus*. Although inbreeding is predicted to reduce genetic variation and hence the ability to adapt to environmental changes, regular inbreeding does allow species to fix favorably adapted gene complexes within the clonal line (Nur 1971; Lynch 1984; Crow 1988; Weider 1993). These favorable gene complexes will not be lost by recombination in closely inbred lineages until outbreeding eventually occurs. The potential for the development of adaptive fixed coadapted gene complexes has been illustrated recently by the demonstration of outbreeding depression in an introduced *X. germanus* population (Peer and Taborsky 2005).

Loxdale et al. (2011) argue strongly that ecological generalism in insects is an “evolutionary improbability.” They advocate strong skepticism about claims of generalism, citing numerous recent cases where “generalists” are found to be complexes of more narrowly adapted cryptic species. The *X. morigerus* in Costa Rica are clearly one species. We have only tested two of many genotypes found worldwide, and for a limited range of resource variation: that these two seem to be host generalists does not mean that all genotypes are. The invasive *X. morigerus* is perhaps the most abundant ambrosia beetle in primary as well as secondary wet tropical forests of Costa Rica: our findings, as limited as they are, give a clear indication that an important component of this success is the presence of GPGs (Table 4).
